# Gender-based harassment in Swedish workplaces and alcohol-related morbidity and mortality: A prospective cohort study

**DOI:** 10.5271/sjweh.4101

**Published:** 2023-09-01

**Authors:** Katrina J Blindow, Emelie Thern, Julio C Hernando-Rodriguez, Anna Nyberg, Linda L Magnusson Hanson

**Affiliations:** 1 Institute of Environmental Medicine, Karolinska Institutet, Stockholm, Sweden.; 2 Swedish Secretariat for Gender Research, Gothenburg University, Gothenburg, Sweden.; 3 Stress Research Institute, Department of Psychology, Stockholm University, Stockholm, Sweden.; 4 Department of Public Health and Caring Sciences, Uppsala University, Uppsala, Sweden.

**Keywords:** alcohol use disorder, client, co-worker, coping, hostility, mistreatment, occupational health, psychosocial work environment, sexism, sexual harassment, Sweden

## Abstract

**Objective:**

The study investigated experiences of different types of work-related gender-based harassment (GBH), specifically sexual and gender harassment, as risk factors for alcohol-related morbidity and mortality (ARMM).

**Methods:**

Information about experiences of (i) sexual harassment (SH-I) and (ii) gender harassment (GH-I) from inside the organization and (iii) sexual harassment from a person external to the organization (SH-E) were obtained from the Swedish Work Environment Survey 1995–2013, a biannual cross-sectional survey, administered to a representative sample of the Swedish working population. The survey responses from 86 033 individuals were connected to multiple registers containing information about alcohol-related diagnoses, treatment, or cause of death. Cox proportional hazard models were fitted to assess hazard ratios (HR) of incident ARMM during a mean follow-up of eight (SH-I and GH-I) and ten (SH-E) years.

**Results:**

A higher prospective risk estimate of ARMM was found among participants who reported experiences of SH-E [HR 2.01, 95% confidence interval (CI) 1.61–2.52], GH-I (HR 1.33, CI 1.03–1.70), or SH-I (HR 2.37, CI 1.42–3.00). Additional analyses, distinguishing one-time from reoccurring harassment experiences, indicated a dose–response relationship for all three harassment types. Gender did not modify the associations. Under the assumption of causality, 9.3% (95% CI 5.4–13.1) of the risk of ARMM among Swedish women and 2.1% (95% CI 0.6–3.6) among Swedish men would be attributable to any of the three types of GBH included in this study.

**Conclusions:**

Experiences of GBH in the work context may be a highly relevant factor in the etiology of ARMM.

As in many other countries, the #MeToo movement resonated strongly in Sweden. People (mostly women) from a wide variety of employment sectors started petitions, giving testimony of the sexist offences they had endured in their working lives and demanding measures to ensure a safe work environment (1). Here, we refer to offensive behavior that the affected attribute to their gender as gender-based harassment (GBH), including sexual harassment and non-sexualizing, sexist experiences. The prevalence of work-related GBH has continuously been documented in the Swedish Work Environment Reports. During 1999–2013, about 18% of women and 6% of men reported at least one work-related experience of sexual harassment or non-sexualizing harassment based on their gender in the past 12 months (2). Still, the individual and societal harm that can be attributed to experiences of work-related GBH is not yet fully understood or recognized as an occupational health hazard (3).

GBH violates workplace rules of mutual respect, insults employees’ professional identities, and disturbs their work performance (4). It may further question a person’s belonging in the occupation (5), and may in some cases constitute a form of bullying (6). Victimized individuals have reported immediate or delayed psychological responses of anxiety and anger as well as self-blame and feelings of isolation (7, 8). Studies have repeatedly found a prospective association of the exposure to sexual harassment with diminished mental health (7, 9–12). Recently, in a different sample from the same study population, we found an excess risk of psychotropic medication use among participants who had experienced sexual or gender harassment (13). Less attention has been given to the effect of work-related GBH on physical health and health behaviors (14). Alcohol consumption has the potential to cause tremendous mental and physical harm as well as put others at risk (15, 16) and is widely recognized as a leading cause of ill health and mortality (17).

Work-related GBH may increase the risk of harm from alcohol consumption when alcohol is used to self-medicate emotional distress (18). A study on US university employees supported the hypothesis that people use alcohol to cope with the distress from workplace harassment (19). Successive longitudinal studies on this cohort confirm an increase of self-reported drinking behaviors or drinking motives known to be associated with alcohol-related problems among the exposed. Follow-up studies suggest a greater effect when harassment persists over time (20, 21) and a lingering effect after it has ceased or the affected has retired (22). However, one study indicated gender differences in the causal direction; harassment predicted later drinking outcomes among women, while drinking behavior predicted later harassment among men (23). A study on a US national sample on the other hand found a prospective association of work-related sexual harassment with self-reports of problematic alcohol use only among men (24). Also, surveys about alcohol consumption are prone to desirability and recall bias (25) and non-response bias (26–28). We overcome these issues by connecting survey responses about work-related GBH to register data of diagnoses and treatment as well as death records as indicators of alcohol-related morbidity and mortality (ARMM). Also, with this approach, we take the research field forward, as alcohol-related diagnoses and treatment are indicative of manifested harm due to alcohol use rather than merely alcohol consumption.

A challenge when studying work-related GBH is the diversity in definitions and measurement instruments (29–31). Here, we investigate two specific forms of GBH: (i) sexual harassment, defined as unwanted advances and offensive remarks of a sexual nature and (ii) gender harassment, defined as non-sexualizing conduct, such as sexist remarks in general or concerning the work context and other disrespectful behavior that the affected consider to be based on their gender. Moreover, we differentiate between harassment exposures from a person inside the organization (ie, superiors or colleagues) versus from a third party, who is external to the organization (eg, clients or customers). The institutional relationship between the harasser and the affected may be crucial for the nature and consequences of GBH. It can be reasoned that harassment from a member of the organization is more problematic due to the higher need for continued cooperation with the harasser (10). On the other hand, third-party harassment is highly normalized in some occupations (32) and possibly more difficult to protect oneself against due to the high prevalence in these occupations (33). Only few studies investigating the prospective association of GBH with health outcomes have made this distinction, and the results have been mixed (6, 10, 12, 13).

Here, we investigate sexual and gender harassment from inside the organization (S&GH-I) and sexual harassment from a person, who is external to the organization (SH-E) as risk factors for the occurrence of ARMM, as indicated by the incidence of an alcohol-related diagnosis, treatment, or cause of death in Swedish registers.

## Methods

### Study population

**Figure 1:**
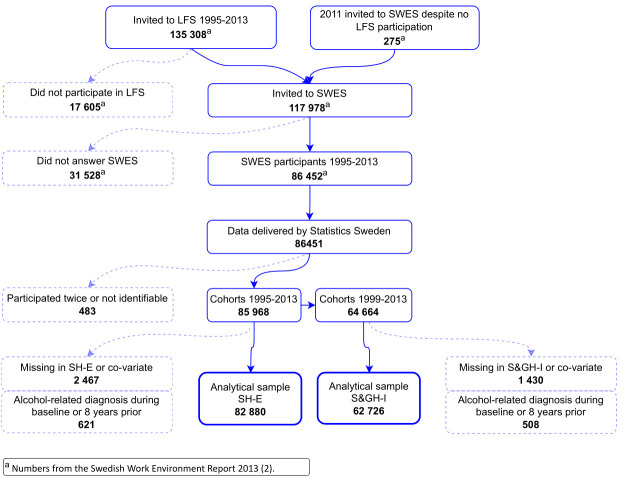
Flow chart of study participants.

The study population comprised all participants of the Swedish Work Environment Survey (SWES) from 1995 to 2013 (N=86 452) (see figure 1 for a flow chart of study participants). SWES is a cross-sectional survey, conducted biannually by Statistics Sweden, building on the Labor Force Survey (LFS), a brief nationwide telephone-supported interview of 15- to 74-year-olds concerning their recent employment situation. The SWES population is a selected subsample of LSF participants, who fulfilled the eligibility criteria of being 16–64 years old, in paid work (≥1 hour/week) and not absent from work in the three months before data collection. Non-participation in the LFS interviews increased from 13% to 33% and in the subsequent SWES from 23% to 51% between 1995 and 2013. The distribution of gender, age, educational level and income in SWES participants is fairly representative of the general population, while individuals born outside Sweden are underrepresented (33).

Here, two analytical samples were defined, one for S&GH-I, including the cohorts 1999 to 2013 and one for SH-E, including cohorts from 1995 to 2013. We excluded individuals with reused identification numbers (N=65), with missing data in the exposures (<2%) or the covariates in the full models (<2%) and individuals who had received an alcohol-related diagnosis or treatment eight years prior to their SWES participation or during that year (<1%). This resulted in 62 726 individuals in analysis of S&GH-I and 82 880 in the analysis of SH-E. Ethical approval for this study was attained from the Regional Research Ethics Board in Stockholm (document numbers: 2012/373- 31/5, 2013/2173–32, 2015/2187–32, 2015/2298–32, and 2017/2535-32). SWES participants received written information that returning the survey indicated informed consent.

### Work-related sexual and gender harassment

The SWES contained two items about sexual harassment from 1995 onwards. Respondents were presented with the definition of sexual harassment as "unwanted advances or offensive remarks generally associated with sex" and asked, if they are subjected to this by (a) superiors or colleagues (SH-I) and (b) other people [eg, patients, clients, passengers, students] (SH-E). An item for the assessment of gender harassment from superiors or colleagues (GH-I) was introduced to the survey in 1999, with a description (see supplementary material, www.sjweh.fi/article/4101, S1) and the question "Are you subjected to harassment of this kind in your workplace by superiors or colleagues?". Respondents rated the three questions separately on a seven-point Likert-type scale with values from not at all in the last 12 months to everyday (see S1).

Based on dichotomous variables ("Not at all in the last 12 months" indicating no exposure and any exposure frequency indicating exposure), we combined GH-I and SH-I into one variable S&GH-I with the three categories: (i) no exposure to sexual or gender harassment; (ii) exposure to gender harassment but not sexual harassment; (iii) exposure to sexual harassment (with or without gender harassment). Gender harassment is widely recognized as an integral part of sexual harassment (in our sample >60% of those exposed to SH-I also reported experiences of GH-I). Therefore, and because of low statistical power, a category of SH-I in the absence of GH-I was not coded. The variable for SH-E was used individually and dichotomized with any frequency other than "Not at all in the last 12 months" coded as exposure. In additional analyses, we explored the role of exposure frequency for the association. The three original variables were categorized into three groups: "Not in 12 months", "Once in 12 months", and "Reoccurring". As above, the variable for SH-E was used individually, and GH-I and SH-I were combined to S&GH-I with the category no exposure and four categories for one-time and reoccurring exposure of GH-I and SH-I respectively. To calculate the population attributable risk percentage, we used the variables from the main analysis and additionally combined them into a binary variable for GBH, counting any exposure as exposed versus the reference group that had reported none of the exposures.

### Alcohol-related morbidity and mortality

We identified cases of ARMM based on the diagnostic codes of the International Classification of Diseases (ICD) versions 9 (1987–96) and 10 (from 1997) suggested by Bergman et al (34). The index includes diagnoses such as eg, alcohol dependency, alcoholic liver disease or acute intoxication (see S2 for diagnostic codes). Information about the date (year and month) and diagnosis were obtained from the National Patient Register (inpatient care from 1987 and outpatient care from 2001), the Social Insurance Agency’s MiDAS register (Sickness Absence Benefits and Disability Pension from 2000), and the Cause of Death register (available since 1961). Since pharmacotherapy is recommended with highest priority for patients with alcohol use disorder (35) and mostly prescribed through primary care (36) in Sweden, we also followed the advice of the Swedish Public Health Agency (37) and identified cases based on a record of pharmacotherapy for alcohol dependency (see S2 for Anatomical Therapeutic Chemical codes) in the Prescribed Drug Register (medication distributed at Swedish pharmacies from mid-2005). In the analyses, data from the different registers were combined into one variable based on the first incident diagnosis or drug purchase. Maximum follow-up was two years (survey participation 2013) to 20 years (survey participation 1995) for SH-E and 16 years (survey participation 1999) for S&GH-I.

### Covariates

We determined potential confounders based on previous research. The variables described henceforth were included in the final models, all referring to the registered status at baseline, according to the Swedish Longitudinal Integrated Database for Health Insurance and Labour Market Studies. Gender was available as the registered identity woman or man, country of birth as “Nordic”, “European”, or “other”, and civil status as “married”/“unmarried”. We grouped age into the categories 16–25, 26–35, 36–45, 46–55, 56–65 years, and attained level of education as ≤12, 13–14, and ≥15 years, divided income into quintiles, and classified participants’ baseline place of residence with a classification available for 2017 as “city”, “urban area” and “rural area”. Further, we used the Patient Register to determine diagnosis of a mental disorder in the eight years prior to the year of survey participation [ICD-9: 290-319 (excluding 303 & 305.0) and ICD-10: F01-F99 (excluding F10)] as to account for the complex, reciprocal relationship between alcohol use disorder and other psychiatric disorders (38).

### Statistical analyses

We performed Cox proportional hazards analyses. Person-time was calculated from January of the year following SWES participation until the incident of an ARMM (failure event), emigration, the year of a participant’s 68^th^ birthday, death (not alcohol-related) or December 2015, whichever occurred first. The proportional hazard assumption was tested with Schoenfeld residuals and “log–log” plots, with no deviations. We performed the Cox regression analyses for the exposure to S&GH-I and the exposure to SH-E separately. For each exposure, we fitted models, adding covariates sequentially, beginning with the crude model, adding the time-stable characteristics survey year, age, gender and country of birth, further adding attained level of education, income, civil status, place of residence, and finally adding prior diagnosis of a mental disorder. In addition, birth cohort, occupation, employment sector, job control and authority were considered, but omitted as inclusion did not affect estimates noteworthy or compromised model fit.

In additional analyses, we distinguished one-time from reoccurring exposure. Further, we conducted two sensitivity analyses. To account for the delay between a medical diagnosis and alcohol-related psychosocial problems that may influence exposure to harassment or the perception of experiences as harassment (reverse causality), we applied a one-year time-lag to the beginning of follow-up and excluded individuals with ARMM in the first year after baseline. Also, since acute alcohol intoxication (F10.0) is mostly experienced only once and predominantly at a young age, in contrast to other alcohol-related diagnoses that indicate longer episodes of harmful alcohol use (34), we conducted analyses, excluding acute alcohol intoxication from the outcome measure. Furthermore, analyses were performed stratifying by gender identity, and interaction on the multiplicative scale was tested. Finally, we used the Stata post estimation command punafcc to calculate the population attributable risk percentage, the proportion of ARMM among women and men that would be attributed to work-related GBH under the assumption of causality and the absence of residual confounding or other sources of bias. All analyses were performed in Stata version 16.1 (IBM Corp, Armonk, NY, USA).

## Results

**Figure 2:**
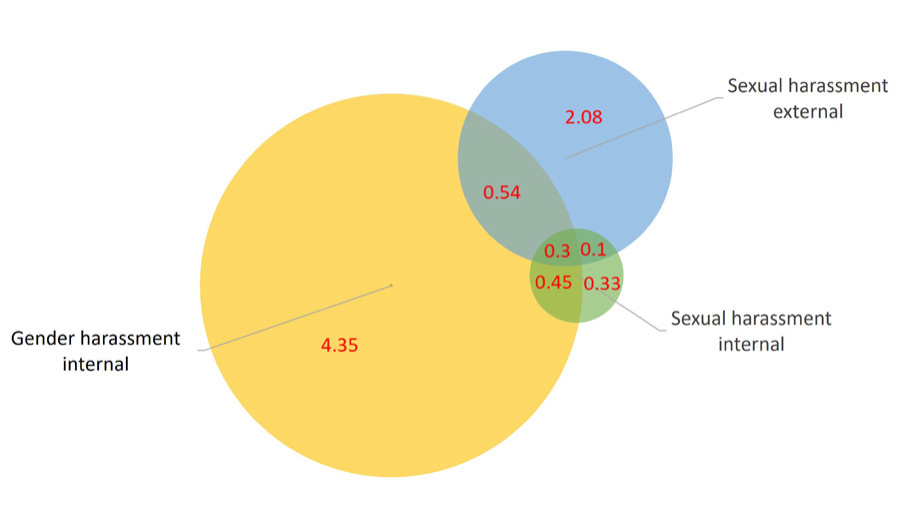
Percent of individuals reporting one or more of the exposures (SWES 1999–2013, N=62 726).

The distribution of the study variables is presented in table 1 and prevalence of the exposures in figure 2. Overall, 2633 (6.1%) women and 523 (1.3%) men reported SH-E; 3665 (11,1%) women and 1137 (3.8%) men reported GH-I; and 725 (2.2%) women and 283 (1%) men reported SH-I. Of those participants experiencing SH-I, 63.7% also experienced GH-I and 33.5% SH-E (not shown). Individuals reporting exposure differed from the total study population in several characteristics, and the patterns differed between the specific exposures. Unmarried participants and those born outside Sweden, particularly those born outside Europe had higher prevalence of all exposures. Among participants reporting SH-E and SH-I, the proportion of young persons (16–25) was higher and older persons (46–65 years) lower. Among participants reporting GH-I, the proportion of 26–45-year-olds was slightly higher. Among those exposed to SH-E and to a lesser extent also among those exposed to SH-I, low-income groups were overrepresented, while GH-I was mostly reported by individuals with a median income (3^rd^ quartile). Furthermore, GH-I and SH-I was most common among participants living in a metropolitan area, and – in all exposed groups – more individuals had received a diagnosis for a mental disorder compared to the whole study population. The most common incidences indicating ARMM among the unexposed and exposed were the diagnosis of a mental and behavioral disorder due to alcohol use (F10) in the patient register and the initiation of pharmacotherapy of alcohol dependency. Alcohol-related somatic diagnoses and benefits (sickness absence or disability) were rare, and no alcohol-related deaths were the first registered incidence in the exposed groups during follow-up (not shown).

**Table 1:** Distribution of study variables among individuals, alcohol-related morbidity and mortality (ARMM) cases and those exposed to external sexual harassment (SH-E) and sexual and gender harassment inside the organization (S&GH-I).

		Sample SH-E N=82 880						Sample S&GH-I N=62 726		
		All		ARMM Cases		SH-E exposed		GH-I exposed		SH-I exposed
		%		%		%		%		%
Gender										
	Woman	52.2		34.6		83.4		76.8		71.9
	Man	47.8		65.4		26.6		23.2		28.1
Age (years)										
	16–25	8.4		8.4		18.1		8.2		17.4
	26–35	20.6		18.4		30.1		24.1		34.7
	36–45	25.6		29.9		24.5		27.8		24.6
	46–55	27.5		31.9		19.8		25.8		16.5
	56–65	17.9		11.3		7.4		14.0		6.8
Country of birth										
	Nordic	92.2		91.4		91.2		90.5		89.8
	European	5.9		7.3		6.1		6.5		7.1
	Other	1.9		1.3		2.7		3.0		3.1
Civil status										
	Unmarried	48.9		68.9		63.8		56.0		70.5
	Married	51.1		41.1		36.2		44.0		29.5
Education (years)										
	≤12	44.1		56.9		37.3		28.7		29.2
	13–14	19.1		16.6		24.1		19.5		26.1
	>14	36.8		26.5		38.6		51.8		44.7
Income (quintiles)										
	1^st^	6.4		9.6		9.8		4.7		7.3
	2^nd^	22.8		24.7		35.6		23.0		24.5
	3^rd^	24.7		22.1		29.4		27.3		27.1
	4^th^	23.7		23.2		17.1		23.9		23.9
	5^th^	22.4		20.4		8.1		21.1		17.2
Place of residence										
	City	30.4		36.9		30.1		36.2		37.9
	Urban area	40.1		37.2		40.7		37.6		37.6
	Rural area	29.5		25.9		29.3		26.2		24.5
Mental disorder										
	No prior diagnosis	98.1		95.3		96.5		96.4		96.7
	Prior diagnosis	1.9		4.7		3.5		3.6		3.3

For the analysis of an association between SH-E and ARMM, the study participants were followed for 889 120 years (mean 10.7 years,), and 1492 participants experienced the event (1.7 cases per 1000 person years). The Cox regression analysis (table 2) gave an unadjusted hazard ratio (HR) of 1.56 [95% confidence interval (CI) 1.25–1.94]. The full model indicated a twofold risk in those reporting SH-E compared to those not exposed in the last 12 months (HR 2.01, 95% CI 1.61–2.52). This increase in the HR was mostly due to the inclusion of age and gender in the model, while adjustment for the other sociodemographic characteristics and a prior mental health diagnosis attenuated the association only slightly.

**Table 2:** Associations of the exposure to sexual harassment from an external person (SH-E) and sexual and gender harassment from an internal person (S&GH-I) with alcohol-related morbidity and mortality (ARMM). [HR=hazard ratio; CI=confidence interval.]

Exposure		ARMM										
		Exposed		Cases		Crude model		Model 1^a^		Model 2 ^b^		Model 3 ^c^
		N		N		HR (95% CI)		HR (95% CI)		HR (95% CI)		HR (95% CI)
SH-E												
	Not in 12 months	79 724		1407		1		1		1		1
	Exposed	3156		86		1.56 (1.25–1.94)		2.20 (1.76–2.75)		2.04 (1.64–2.55)		2.01 (1.61–2.52)
S&GH-I												
	Not in 12 months	57 550		894		1		1		1		1
	Gender harassment ^d^	4168		67		1.05 (0.82–1.34)		1.37 (1.06–1.76)		1.36 (1.06–1.75)		1.33 (1.03–1.70)
	Sexual harassment ^e^	1008		26		1.63 (1.08–2.35)		2.10 (1.42–3.11)		2.01 (1.36–3.97)		2.37 (1.42–3.00)

^a^ Adjusted for wave, age, gender and country of birth. ^b^ Model 1 + education, income, civil status and living area at baseline. ^c^ Model 2 + mental health diagnosis in the past 8 years. ^d^ Gender harassment only, cases of sexual harassment excluded. ^e^ Sexual harassment without or with gender harassment.

For the analysis of an association between S&GH-I and ARMM, study participants were followed for 515 729 years (mean 8 years), and 987 participants experienced the event (1.9 cases per 1000 person years). The Cox regression analysis (table 2) gave a HR of 1.05 (95% CI 0.82–1.34) for GH-I and HR 1.63 (95% CI 1.08–2.35) for SH-I. Similar to the analysis of SH-E, adjustment for age and gender increased the HR, while adjustment for further characteristics made little difference. An exception was SH-I, where adjusting for a prior mental health diagnosis increased the HR considerably. In the full model, the HR for GH-I was 1.33 (95% CI 1.03–1.70) and 2.37 (95% CI 1.42–3.00) for SH-I.

Additional analyses with separate categories for one-time and reoccurring exposure consistently resulted in higher HR among individuals reporting reoccurring exposure than those reporting one experience in the past 12 months (table 3). Sensitivity analyses with the fully adjusted models confirmed the robustness of the main results; HR changed slightly but remained statistically significant. Leaving a one-year time-lag between survey participation and the beginning of follow-up gave slightly lower estimates for SH-E and SH-I and a higher estimate in GH-I (see S3). Exclusion of incidences of acute alcohol intoxication (176 cases) from the outcome measure gave slightly higher excess risks among those exposed to SH-E and GH-I and a slightly lower association of SH-I with ARMM (see S4).

**Table 3:** Associations of the exposure to sexual harassment from an external person (SH-E), gender harassment from inside the organization (GH-I) and sexual harassment from inside the organization (SH-I) with alcohol-related morbidity and mortality (ARMM). Adjusted for age, gender, country of birth, education, income, civil status, living area, mental health diagnosis at baseline and 8 years prior. [HR=hazard ratio; CI=confidence interval.]

Exposure		ARMM		
		Exposed	Cases	HR (95% CI)
		N	N	
SH-E				
	Not in 12 months	79 724	1407	1
	Once in 12 months	2167	48	1.66 (1.24–2.22)
	Reoccurring	989	38	2.77 (2.00–3.84)
G&SH-I				
	Not in 12 months	57 550	894	1
	GH-I once in 12 months ^a^	2793	37	1.15 (0.82–1.60)
	GH-I reoccurring ^a^	1336	30	1.65 (1.14–2.38)
	SH-I once in 12 months ^b^	654	12	1.50 (0.85–2.67)
	SH-I reoccurring ^b^	354	14	2.88 (1.69–4.89)

^a^ Gender harassment only, cases of sexual harassment excluded. ^b^ Sexual harassment without or with gender harassment.

**Figure 3:**
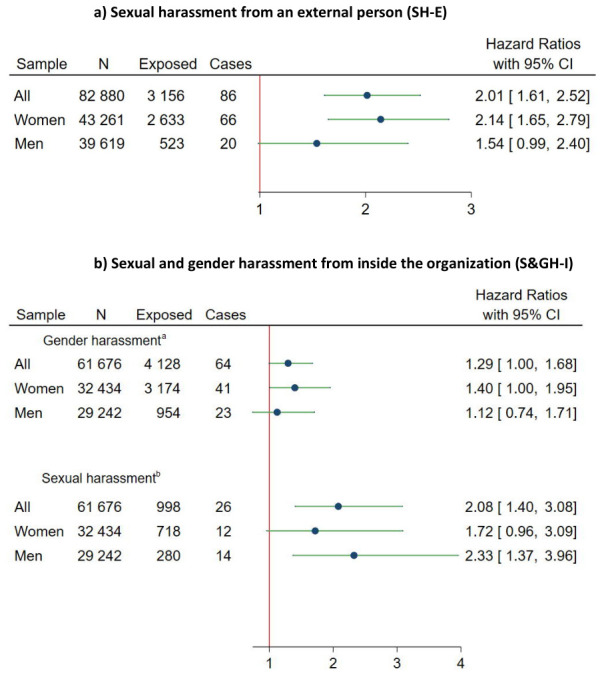
Associations of the exposure to a) sexual harassment from an external person (SH-E) and b) sexual and gender harassment from an internal person (S&GH-I) with alcohol-related morbidity and mortality (ARMM). Adjusted for age, gender, country of birth, education, income, civil status, living area, mental health diagnosis at baseline and 8 years prior. ^a^ Gender harassment only, cases of sexual harassment excluded. ^b^ Sexual harassment with or without gender harassment.

Interaction analyses indicated no difference in the association between the exposures and ARMM between women and men (P-value Wald test: SH-E=0.12, S&GH-I=0.43). Moreover, no clear pattern could be seen in the gender-stratified analyses (figure 3), though the HR was lower among men than women for SH-E and GH-I, but higher for SH-I. The population attributable risk of ARMM due to GBH was 9.3% (95% CI 5.4–13.1) [5.7 (95% CI 2.4–8.9) for S&GH-I and 6.8 (95% CI 5.2–8.4) for SH-E) among women and 2.1 (95% CI 0.6–3.6) (1.5 (95% CI 0.7–3.0) for S&GH-I and 0.7 SH-E (95% CI 0.1–1.4)] among men.

## Discussion

### Main findings

In this prospective cohort study, we found an excess risk of ARMM among Swedish women and men who experienced sexual or gender harassment in their work context. While the associations between the different types of GBH and ARMM were similar among women and men, GBH accounted for a greater share of the ARMM cases among women.

### Comparison to prior studies and interpretation

To our knowledge, this is the first study to investigate the association of sexual and gender harassment with a register-based measure of harm from alcohol use. All prior studies that we are aware of (19–24) investigated the association between sexual harassment experiences and actual self-reported drinking behaviors and motives. Moreover, while we relied on participants’ self-labelling of experiences as harassment, the surveys used in prior studies presented participants with a battery of behaviors and defined sexual harassment cases with a cut-off, varying between one and more than one experiences. This gave considerably higher prevalence (≥30%) and lower gender differences in prevalence in the other studies. Still, our results are mostly in line with those of the former studies in demonstrating a prospective relationship between work-related sexual harassment and alcohol use (19–24). The excess risk of ARMM remained over a long time after the reported harassment experiences. This is in line with the findings of McGinley et al (20) and Richman et al (22), who also found an association over longer time-periods. Such a prolonged effect may manifest through many pathways. GBH can initiate a downward spiral of stress proliferation (7), as victims often find themselves unprotected by their employers (39–41), and their efforts to end the harassment are often ineffective or can even backfire (21, 42, 43). Therefore, victims are at risk of experiencing institutional betrayal on top of the initial insult (44), having to exit the workplace under unfavorable conditions (33, 45) and suffering long-term financial losses (46).

We are the first to distinguish between one-time and reoccurring harassment and found indications of a dose–response relationship in all three GBH types. This confirms the assumption of a high relevance of pervasiveness of the harassment (47) and suggests that mostly individuals who were exposed repeatedly subsequently experienced harm from their alcohol use. Furthermore, no prior study investigated gender harassment in relation to alcohol outcomes or specified whether harassment stemmed from a person inside or outside the work organization. Our results suggest a higher vulnerability of people to sexual harassment than gender harassment. In earlier studies, we found no such differences in effect size between sexual and gender harassment in the prospective relation to long-term sickness absence (48) or psychotropics use (13). The association of SH-E and SH-I with ARMM was similar, though, due to fewer cases of SH-I, this estimate was less precise. Prior studies found a stronger association of SH-I than SH-E with depressive disorder (10) and sickness absence (6). However, studies based on the same cohort as ours found no distinct difference between SH-I and SH-E in relation to prospective psychotropics use (13), but a stronger association of SH-E than SH-I with suicide (12). Given that the Swedish work environment and antidiscrimination laws do not cover misconduct from third parties to the same extent as from inside the organization (49) and that interventions need to focus on different occupational contexts for targeting SH-I or SH-E (33), further investigation into the matter may be motivated.

Prior studies have been inconclusive regarding the role of gender in the association between work-related sexual harassment and alcohol use. Two studies found associations among both women and men (19, 21), one only among men (24) and one a prospective relation only among women, and a reverse relation among men, alcohol use predicting sexual harassment (23). We had low statistical power to investigate gender differences and found no pronounced differences in the association of S&GH-I or SH-E with ARMM. We found different contributions of work-related GBH on the population risk of ARMM among women and men, though. In part, this is explained by the higher exposure among women. In part, it may be explained by differences between the drinking motives of Swedish women and men. In a report by Ramstedt et al (16), coping motives generally played a minor role, and were slightly more often stated by men (16). More common were enhancing motives, ie, drinking to feel good, and they were significantly more relevant for men than women. Both coping and enhancing motives were highly associated with alcohol-related problems (DSM-5 criteria). Men also scored considerably higher on confirmative and social motives, indicating that alcohol is a “gender symbol” in contemporary Sweden, confirming with masculinity and possibly conflicting with femininity (50, 51). Overall, our results indicate that for women presenting with harm from alcohol use, work-related GBH may be an important factor in the disease etiology, while other factors are more relevant among men, who generally have a higher disease prevalence.

### Strengths and limitations

As far as we are aware, previous studies on the relation of GBH and alcohol use were only conducted on two American cohorts. This is the first study to investigate the association between workplace sexual and gender harassment and ARMM on a large sample that is approximately representative of the Swedish working population. The exposure items were placed in the middle of a survey about the general work environment, containing no questions about alcohol use. This is a major strength, as it limits differential selection into the study regarding exposure or outcome status at baseline. Using administrative registers to identify ARMM avoided challenges with self-reported outcome measures, and allowed for a continuous, long follow-up. This being said, the assessment of GBH based on respondents’ self-labelling is rather conservative, as only a minority of cases that are identified by researchers based on behavior-based measures tend to be recognized by study participants as harassment (52, 53). Also, we could not determine the onset of the harassment or the nature and severity of the experience. The unclear exposure onset and the considerable time that can pass between alcohol-related harm and a diagnosis complicate determining the sequence of events. On the one hand, excluding individuals with an alcohol-related diagnosis before baseline and adjusting for prior mental health diagnoses could have been an overcontrol when the harassment had been ongoing for several years. On the other hand, the results could be spurious with reverse causality, if undiagnosed alcohol-related psychosocial problems influenced the risk of harassment experiences. We responded to the former by also presenting the results without adjustment for prior mental health disorders and the latter with the sensitivity analysis applying a one-year time-lag to the beginning of follow-up. Furthermore, with this study design, we cannot rule out confounding by personality traits or biographic influences that may affect people’s propensity to experience or identify harassing conduct and contribute to their risk of ARMM or their help seeking behavior. Particularly parental socioeconomic situation (54), mental health, and alcohol use (55) could not be considered. However, we did adjust for study participants’ mental health diagnoses, particularly alcohol-related diagnoses prior to exposure assessment, and had access to vast information about their baseline familial, economic, and working situation.

### Concluding remarks

The results of this study suggest that work-related experiences of GBH, both of a sexual and a non-sexual nature, may contribute considerably to the etiology of ARMM among women and to some extent men. Particularly people experiencing sexual harassment and those repeatedly exposed to harassment may subsequently experience harm from their alcohol use, regardless if the harassment stems from a member of their organization or a third party. In high-prevalence occupations, GBH qualifies as an occupational health hazard that needs to be approached systematically by employers, and employer responsibility for protection against third party harassment may need specification.

## Supplementary material

Supplementary material
