# Supplementary material for: Gender-based harassment in Swedish workplaces and alcohol-related morbidity and mortality: A prospective cohort study
Source: Scand J Work Environ Health. 2023 Aug 29;49(6):395–404. doi: 10.5271/sjweh.4101 (PMC10782510; doi:10.5271/sjweh.4101)
Supplement: Supplementary material [file SJWEH-49-395-S001.pdf]

Gender-based harassment in Swedish workplaces and alcohol-related morbidity and mortality: A prospective cohort study[1]

by Katrina J Blindow, MPH,[2]Emelie Thern, PhD, Julio C Hernando-Rodriguez, PhD, Anna Nyberg, PhD, Linda L Magnusson Hanson, PhD

1. Correspondence to: Katrina J Blindow, Institute of Environmental Medicine, Karolinska Institutet, 171 77 Stockholm, Sweden. [E-mail: katrina.blindow@ki.se]
2. Supplementary material

S1 Exposure measures in the Swedish Work Environment Survey

Sexual harassment

"In the following two questions, sexual harassment refers to unwanted advances or offensive remarks generally associated with sex.

Are you subjected to sexual harassment in your workplace from...

...supervisors or colleagues?

...other persons (e.g. customers, patients, clients, passengers, students)? "

Gender harassment

"The next question concerns whether you have experienced conduct (other than that described above) which is based on your gender and that hurts your integrity or is degrading. This can be for example, condescending and ridiculing remarks about men or women in general or in the context of your profession. It can also mean that somebody does not take notice of you or of your contributions because of your gender.

Are you subjected to harassment of this kind in your workplace by superiors or colleagues?"

Scale provided for each item and categories in the variables used in the analyses

Every day	A couple days per week (1 day of 2)	One day per week (1 day of 5)	A couple days per month (1 day of 10)	A few times in the last 3 months	Sometime in the last 12 months	Not in the last 12 months

Dichotomous	Exposed						Not in 12 months
Three categories	Reoccurring					Once in 12 months	Not in 12 months
Original scale	Every day	A couple days per week (1 day of 2)	One day per week (1 day of 5)	A couple days per month (1 day of 10)	A few times in the last 3 months	Sometime in the last 12 months	Not in the last 12 months

S2 ICD-9, ICD-10 and ATC codes included in the outcome measure ARMM

Cases of ARMM were identified in the National Patient Register, Social Insurance Agency's MiDAS register, and Cause of Death register if the primary or secondary diagnosis was registered with at least one of the ICD-9 codes 303, 305A, 357F, 425F, 535D, 571B, 571D, 571C, 571A, 980A, 980X, 790D, 977D, V79B or at least one of the ICD-10 codes F10.0, E24.4, G62.1, I42.6, K29.2, G31.2, G72.1, K70.1, K70.9, K70.3, K70.0, K70.2, K70.4, K85.2, K86.0, T51.0, T51.9, R78.0, O35.4, Y57.3, Z50.2, Z71.4, Z72.1, X65.

Cases of pharmacotherapy for alcohol dependence were identified in the Prescribed Drug register, if at least one of the Anatomical Therapeutic Chemical codes N07BB01, N07BB03, N07BB04, N07BB05 was registered.

S3 Sensitivity analysis applying a 1-year time-lag after exposure assessment

Associations of the exposure to sexual harassment from an external person (SH-E) and sexual and gender harassment from an internal person (GBH-I) with ARMM**.** Adjusted for age, gender, country of birth, education, income, civil status, living area, mental health diagnosis at baseline and 8 years prior.

Exposure		ARMM	
	Exposed	Cases	
	n	n	HR (95% CI)
**SH-E**			
Not in 12 months	79 646	1 329	1
Exposed	3 147	77	1.92 (1.51-2.42)
**S&GH-I**			
Not in 12 months	57 479	823	1
Gender harassment [a]	4 164	63	1.37 (1.05-1.77)
Sexual harassment [b]	1 006	24	2.03 (1.35-3.06)

[a] Gender harassment only, cases of sexual harassment excluded

[b] Sexual harassment without or with gender harassment

S4 Sensitivity analysis, excluding acute alcohol intoxication (F10.0) from the outcome measure

Associations of the exposure to sexual harassment from an external person (SH-E) and sexual and gender harassment from an internal person (S&GH-I) with ARMM excluding acute alcohol intoxication (F10.0). Adjusted for age, gender, country of birth, education, income, civil status, living area, mental health diagnosis at baseline and 8 years prior.

Exposure		ARMM	
	Exposed	Cases	
	n	n	HR (95% CI)
**SH-E**			
Not in 12 months	79 724	1 228	1
Exposed	3 156	75	2.12 (1.67-2.69)
**S&GH-I**			
Not in 12 months	57 550	773	1
Gender harassment [a]	4 168	63	1.47 (1.13-1.90)
Sexual harassment [b]	1 008	23	2.19 (1.45-3.33)

[a] Gender harassment only, cases of sexual harassment excluded

[b] Sexual harassment without or with gender harassment
